# Fertility Preservation in Female Cancer Patients

**DOI:** 10.5402/2012/807302

**Published:** 2012-01-26

**Authors:** Chung-Hoon Kim, Gyun-Ho Jeon

**Affiliations:** ^1^Division of Reproductive Endocrinology and Infertility, Department of Obstetrics and Gynecology, Asan Medical Center, College of Medicine, University of Ulsan, 388-1 Poognap-dong, Songpa-gu, Seoul 138-736, Republic of Korea; ^2^Division of Reproductive Endocrinology and Infertility, Department of Obstetrics and Gynecology, Haeundae Paik Hospital, College of Medicine, Inje University, Busan, Republic of Korea

## Abstract

With improved survival rates among cancer patients, fertility preservation is now being recognized as an issue of great importance. There are currently several methods of fertility preservation available in female cancer patients and the options and techniques via assisted reproduction and cryopreservation are increasing, but some are still experimental and continues to be evaluated. The established means of preserving fertility include embryo cryopreservation, gonadal shielding during radiation therapy, ovarian transposition, conservative gynecologic surgery such as radical trachelectomy, donor embryos/oocytes, gestational surrogacy, and adoption. The experimental methods include oocyte cryopreservation, ovarian cryopreservation and transplantation, in vitro maturation, and ovarian suppression. With advances in methods for the preservation of fertility, providing information about risk of infertility and possible options of fertility preservation to all young patients with cancer, and discussing future fertility with them should be also considered as one of the important parts of consultation at the time of cancer diagnosis.

## 1. Introduction

As a result of a remarkable improvement in the survival rates of cancer patients, there has been an increased interest in the long-term effects of cancer treatment on quality of life. In particular, infertility is one of the major sequelae of caner therapy and may be considerable distress to cancer survivors. In female patients, risk of menopause-related complication and infertility at a very young age due to cancer treatment may be more devastating and be considered as a loss of their essential femininity [1]. Even in patients who were not sterilized after chemotherapy or radiation therapy, increased risks of complications during pregnancy, such as early pregnancy loss, premature labor, and low birth weight, have been reported [2].

Although infertile patients after cancer treatment can become parents with adoption or embryo or oocyte donation, many would prefer to have biological offspring [3], thus the demands from such patients and the creation of various options and techniques for fertility preservation by reproductive specialists are increasing. Several national organizations in USA have recently published fertility-preservation guidelines: the President's Cancer Panel (2004), the American Society of Reproductive Medicine Ethics Committee (2005), and the American Society of Clinical Oncology (2006) [4]. Some methods such as embryo cryopreservation, ovarian transposition are considered standard practice but some other methods should be considered investigational. Choosing the most appropriate ones among these methods depends on the individual's status such as the type of cancer, the variations of cancer treatment, the time available before onset of treatment, the patient's age, and the partner status.

This paper describes the risks for infertility, current and emerging options for the fertility preservation in female cancer, patients and the clinical/ethical issues with respect to fertility.

## 2. Effects of Cancer Treatment on Female Fertility

The most common cancers diagnosed in people under the age of 40 are breast cancer, melanoma, cervical cancer, non-Hodgkin's lymphoma, and leukemia [5]. The treatment for these malignancies implies either surgery, radiotherapy, chemotherapy, or a combination, and they can compromise the function of reproductive system.

In the treatment of gynecologic malignancies, the standard treatment includes surgical removal of uterus and ovary, which eliminates the possibility of childbearing [6]. Multiple chemotherapeutic agents for many cancers result in irreversible gonadal damage, which is related to the decline of numbers of primordial follicles as well as a reduction in the numbers of larger maturing follicles [7–9]. The end result of chemotherapy-induced damage is often premature ovarian failure leading to permanent infertility. The risk of ovarian damage caused by the different chemotherapeutic agents is shown in Table 1. Alkylating agents (particularly cyclophosphamide, ifosfamide, nitrosaureas, chlorambucil, melphalan, busulfan, and procarbazine) are associated with the greatest risk of infertility while several agents (methotrexate, fluorouracil, vincristine, bleomycin, and dactinomycin) are associated with a low or no risk of infertility [4, 10].

Radiation also causes a reduction in the primordial follicle pool, and the degree of ovarian damage is dependent on dose and field [11, 12]. Total-body irradiation and pelvic irradiation that includes the ovaries can highly produce permanent ovarian failure, while lesser dose or limited radiation fields not related to ovarian location have less gonadal toxicity [13, 14]. The uterine volume is also affected by irradiation and decreases by 40%. Even if these patients become pregnant, they present with restricted blood flow and impaired uterine growth thus have higher risk of pregnancy complications including early abortion, preterm labor, and low birth weight [15]. Cranial radiation greater than 35 to 40 Gy can impair the hypothalamic pituitary function and cause hypogonadism [16].

The effects of cancer treatment on subsequent ovarian function vary and depend on many factors—drug, dose, size/location of radiation field, method of administration, disease, age, and pretreatment fertility of the patients [4]. Therefore, when estimating the risk of infertility in female cancer patients, the consideration of these factors should be essential. For example, older women have higher risk of ovarian failure after chemotherapy compared with younger patients because of the reduced primordial follicle pool with aging. Patients who resume thier ovarian function following chemotherapy or radiotherapy should be recommended not to delay childbearing for long times, but should be advised not to become pregnant before 6–12 months after treatment because of the toxicity of cancer treatment on oocytes [17].

## 3. Options for Fertility Preservation in Female Cancer Patients

### 3.1. Established Methods of Fertility Preservation (Table 2)

#### 3.1.1. Embryo Cryopreservation

Embryo cryopreservation is the most established option for preserving fertility and is widely used with well-defined success rates [4]. This method is performed with the course of in vitro fertilization procedure involving ovarian stimulation, oocyte retrieval, fertilization. Therefore, it requires 10–14 days from menses for follicular development and necessitate a delay of the chemotherapy initiation. Male partner or sperm donor for embryo creations is also needed. One of the concerns of this treatment is the high serum estrogen concentration during ovarian stimulation in patients with hormone-sensitive tumors such as breast cancer [6, 18]. The use of tamoxifen or letrozole—SERM (selective estrogen receptor modulator) or aromatase inhibitor—for ovarian stimulation to reduce the risk of estrogen exposure revealed no increase of cancer recurrence rates in some studies [19, 20], but larger and long-term follow-up results are needed.

#### 3.1.2. Ovarian Transposition/Radiation Shielding of Gonads

Ovarian transposition (oophoropexy) is a surgical procedure that places the ovaries outside of radiation field to protect them from irradiation. The overall success rate to retain menstrual function and fertility is not much better than about 50% due to scattered radiation and altered blood flow [4]. The success of this procedures depends on the extent of radiation scatter, vascular damage, the age of patients, total radiation dose and whether or not the ovaries are shielded [12, 21]. There is no strong rationale of this procedure when concurrent gonadotoxic chemotherapy is performed with radiation [22].

Radiation shielding of gonads is also a standard method for fertility preservation. Use of shielding during radiotherapy is to reduce scatter radiation to the reproductive organ.

#### 3.1.3. Conservative Gynecologic Surgery

Although the traditional and ultimate treatment of gynecologic malignancies includes surgical removal of, or radiation to uterus and ovary, new approaches have been developed in gynecologic oncologic surgery, focused on the preservation of key reproductive organs. Radical trachelectomy, a surgical removal of the cervix with preservation of the uterus, is a typical established one of the conservative surgery for the fertility preservation [23]. This operation should be restricted to early-stage IA2-IB disease with less than 2 cm in diameter and less than 10 mm invasion [24]. Rates of recurrence are comparable to those treated by means of radical hysterectomy [25]. Spontaneous pregnancies were described up to 70%, although there is increased risk of second trimester pregnancy loss, preterm delivery, and need of the use of assisted reproduction technologies [26, 27].  Table 3 shows current indication and definition of conservative surgery in other gynecologic malignancies [10].

#### 3.1.4. Embryo/Oocyte Donation and Adoption

Although infertile patients after cancer treatment can become parents with embryo/oocyte donation, they would not become biologic mothers. Therefore, one of the ways of the future in oocyte donation may be a donation protocol using enucleated donor oocytes.

### 3.2. The Experimental Methods for Fertility Preservation (Table 4)

The Panel of ASCO (American Society of Clinical Oncology, 2006) recommended that the efforts to preserve fertility using experimental methods should be attempted under institutional-review-board- (IRB-) approved protocols [4].

#### 3.2.1. Oocyte Cryopreservation

Cryopreservation of oocytes can be considered as good alternative methods particularly for single women who do not have a partner or sperm donor. However, unfertilized mature oocytes are more fragile and are easily damaged during the freezing or thawing process, compared with the embryo [28]. However, with recent improvement in freeze-thaw protocols such as vitrification, promising results, more than 60% of mature oocytes surviving after thawing and subsequent fertilization rates comparable with fresh oocytes, were reported [29, 30]. Like with embryo preservation, this option needs ovarian stimulation and harvesting, thus concerns regarding time delay in cancer therapy and risk of exposure to high hormonal level can be raised.

#### 3.2.2. Cryopreservation and Transplantation of Ovarian Tissue

Ovarian tissue cryopreservation requires neither a sperm donor nor ovarian stimulation thus can be the only feasible option for prepubertal girls and be performed immediately after cancer diagnosis. Ovarian tissue is removed via laparoscopy or laparotomy and frozen. After cancer treatment, the ovarian tissue is thawed and reimplanted. Cryopreservation of the primordial follicles in cortical tissue using slow programmed freezing has better results, with up to 65% of survival of follicles, thus is the current widely used protocol for this method [31]. Thawed ovarian tissue can be reimplanted orthotopically or heterotopically, and about 20 cases of autotransplantation of cryopreserved ovarian tissue with 7 live births after orthotopic transplantation have been reported [32–36]. The one concern proposed in the reimplantation of ovarian tissue is the retransmission of malignant cells, although there were no reports of cancer recurrence after ovarian transplantation in the previous reports. Thus the detection of cancer cells in ovarian tissue should be performed to minimize this risk [4, 37].

#### 3.2.3. In Vitro Maturation

In vitro maturation of immature oocytes and fertilization has been tried in some center. Since it does not require standard ovarian stimulation, this can be a useful procedure for female cancer patients who need prompt cancer therapy. Despite the development of culture system permitting accelerated maturation and development of primordial and primary follicles, implantation and pregnancy rates are generally lower than for IVF with mature oocytes [38, 39]. Further advance of culture protocol is needed.

#### 3.2.4. Ovarian Suppression

Ovarian suppression induced by gonadotropin-releasing hormone (GnRH) analogs was expected to offer ovarian protection during chemotherapy, but this method has still insufficient evidence. Although animal studies have shown the protective effect from gonadal damage during chemotherapy [40] and some studies in humans have also suggested a protecive effect of GnRH agonists, these studies are criticized for some methodologic limitations such as retrospective nature, lack of randomization, short duration of follow-up, use of heterogeneous patient groups, and chemotherapy regimen [41–43]. Therefore, the results of large prospective, randomized clinical studies would be needed for defining the effectiveness of this option.

## 4. Clinical and Ethical Issues

In clinical practice physician needs to discuss with cancer patients about infertility as a potential risk of cancer therapy and inform the methods of fertility preservation or refer to reproductive specialist, but many patients have no chance to discuss about the fertility [44]. This seems to be resulted from oncologists' lack of knowledge about fertility preservation methods, prioritizing discussion about life-threatening complications, concern about potential treatment delay, and overestimation of financial cost [45]. Additionally, some ethical issues—the choice of option for fertility preservation among established and experimental methods, consenting problem of patients under the age of 18, delaying of cancer therapy, and disposition of embryos, oocytes, ovarian tissue—can be faced and remained as questions when providing information and practicing of fertility preservation [45].

## 5. Conclusion

With the development of conservative gynecologic surgery and advanced technologies in assisted reproduction and cryopreservation, the options of fertility preservation in female cancer patients are developing and various methods became applicable in many cases. Radical trachelectomy can be performed for early-stage cervical cancer patients. Embyro cryopreservation can be suggested to women with a partner. Oocyte cryopreservation can be useful for young females without partner. Cryopreservation of ovarian tissue is the only option for prepubertal cancer patients and a feasible method for all patients from different age groups. These options should be selected individually considering each patient's status such as age, partner status, medical condition, and other situations. Above all things, discussion with patients about the options for fertility preservation at the time of cancer diagnosis would be the most important task in current circumstances that the practice has not become routine, distinguishing between established and experimental interventions.

## Figures and Tables

**Table 1:** Risks of gonadotoxicity in different chemotherapeutic agents.

High risk	Cyclophosphamide, ifosfamide, nitrosoureas, busulfan, chlorambucil, melphalan, procarbazine
Intermediate risk	Cisplatin, adriamycin
Low or no risk	Methotrexate, fluorouracil, vincristine bleomycin, dactinomycin
Unknown risk	Taxanes, oxaliplatin, irinotecan, monoclonal antibodies, tyrosine kinase inhibitors

**Table 2:** The established methods for fertility preservation.

Option	Embryo cryopreservation	Ovarian transposition /radiation shielding gonad,	Radical trachelectomy	Donor embryos/donor oocytes/gestational surrogacy/adoption
Pubertal status	After puberty	Before or after puberty	After puberty	After puberty
Time requirement	10–14 days from mens/outpatient procedure	In conjunction with radiotherapy/outpatient procedure	In patient surgical procedure	Varies: in conjunction with IVF
Success rates	Approximately 20–33% per transfer	Approximately 50% due to altered blood flow and scattered radiation	No evidence of higher cancer Recurrence rates	Embryo: unknown/oocytes: 40–50%/ surrogacy: 30%

**Table 3:** Conservative surgery in gynecologic malignancies.

Indication	Type of surgery	Definition
Cervical cancer stage 1A2-1B1	Radical vaginal trachelectomy	Laparoscopic pelvic lymphadenectomy, resection of cervix and parametrium
Borderline ovarian tumors FIGO stage I	Unilateral oophorectomy	Removal of the affected ovary
Ovarian epithelial cancer stage I, grade 1	Unilateral oophorectomy	Removal of the affected ovary
Malignant ovarian germ-cell tumor/sex cord-stromal tumors	Unilateral oophorectomy	Removal of the affected ovary
Epithelial adenocarcinoma grade 1, stage 1A	Hormonal treatment with progestational agents for 6 months [12]	Follow-up wit endometrial biopsies every 3 months

**Table 4:** The experimental methods for fertility preservation.

Option	Oocytes cryopreservation	Ovarian tissue cryopreservation and transplantation	In vitro maturation	Ovarian suppression
Pubertal status	After puberty	Before or after puberty	After puberty	After puberty
Time requirement	10–14 days from men /outpatient procedure	Outpatient surgical procedure	2–10 days, outpatient surgical procedure	In conjunction with chemotherapy
Success rates	Approximately 21.6% per transfer	Case reports of 7 live births	Up to 30% per embryo transfer	Conflicting results reported
